# Prophylactic Treatment with Adlay Bran Extract Reduces the Risk of Severe Acute Radiation Dermatitis: A Prospective, Randomized, Double-Blind Study

**DOI:** 10.1155/2015/312072

**Published:** 2015-10-01

**Authors:** Chih-Jen Huang, Ming-Feng Hou, Jung-Yu Kan, Chiung-Hui Juan, Shyng-Shiou F. Yuan, Kuei-Hau Luo, Hung-Yi Chuang, Stephen Chu-Sung Hu

**Affiliations:** ^1^Department of Radiation Oncology, Kaohsiung Medical University Hospital, Kaohsiung 807, Taiwan; ^2^Faculty of Medicine, College of Medicine, Kaohsiung Medical University, Kaohsiung 807, Taiwan; ^3^Department of General Surgery, Kaohsiung Medical University Hospital, Kaohsiung 807, Taiwan; ^4^National Sun Yat-Sen University-Kaohsiung Medical University Joint Research Center, Kaohsiung 807, Taiwan; ^5^Division of Gastrointestinal and General Surgery, Department of Surgery, Kaohsiung Medical University Hospital, Kaohsiung 807, Taiwan; ^6^Cancer Center, Kaohsiung Medical University Hospital, Kaohsiung Medical University, Kaohsiung 807, Taiwan; ^7^Graduate Institute of Clinical Medicine, College of Medicine, Kaohsiung Medical University, Kaohsiung 807, Taiwan; ^8^Translational Research Center, Department of Obstetrics and Gynecology, Kaohsiung Medical University Hospital, Kaohsiung 807, Taiwan; ^9^Institute of Occupational Safety and Health, Department of Public Health, Kaohsiung Medical University, Kaohsiung 807, Taiwan; ^10^Department of Dermatology, Kaohsiung Medical University Hospital, Kaohsiung 807, Taiwan; ^11^Department of Dermatology, College of Medicine, Kaohsiung Medical University, Kaohsiung 807, Taiwan

## Abstract

Acute radiation dermatitis is a frequent adverse effect in patients with breast cancer undergoing radiotherapy, but there are only a small number of studies providing evidence-based interventions for this clinical condition. Adlay is a cereal crop that has been previously shown to have anti-inflammatory and antioxidant properties. In this study, we seek to evaluate the effectiveness of oral prophylactic treatment with adlay bran extract in reducing the risk of severe acute radiation dermatitis. A total of 110 patients with breast cancer undergoing radiotherapy were analyzed. Using a prospective, randomized, double-blind design, 73 patients received oral treatment with adlay bran extract and 37 patients received olive oil (placebo). Treatment was started at the beginning of radiation therapy and continued until the termination of radiation treatment. Our results showed that the occurrence of severe acute radiation dermatitis (RTOG grade 2 or higher) was significantly lower in patients treated with oral adlay bran extract compared to placebo (45.2% versus 75.7%, adjusted odds ratio 0.24). No serious adverse effects from adlay bran treatment were noted. In conclusion, prophylactic oral treatment with adlay bran extract reduces the risk of severe acute radiation dermatitis and may have potential use in patients with breast cancer undergoing radiotherapy.

## 1. Introduction

Radiation therapy is commonly used for the treatment of various types of cancers. It can be used alone or in combination with other forms of treatment (such as surgery and chemotherapy). However, radiotherapy is limited by its potential to cause injury to normal tissues. The skin contains cells with high proliferative rate and is therefore one of the tissues most susceptible to radiation damage. Cells in the skin that are highly radiosensitive include the epidermal basal keratinocytes and hair follicle stem cells [1]. Radiation dermatitis occurs frequently in patients with breast cancer receiving radiotherapy and may limit the duration and total dose of radiation treatment [2, 3].

Radiation-induced skin injury may be categorized as acute or chronic [4]. Acute radiation dermatitis occurs within hours to weeks after initiation of radiation treatment, whereas chronic radiation dermatitis develops months to years after radiotherapy. In this study, we are mainly interested in evaluating acute radiation dermatitis. The clinical features of acute radiation dermatitis include skin erythema, dry desquamation, moist desquamation, and changes in pigmentation. A subset of patients may develop severe forms of radiation dermatitis with skin ulceration and necrosis [2].

Although various topical and oral agents have been used for the prevention or treatment of acute radiation skin reactions, only a small number of controlled trials have been performed, and some of the evidence is conflicting [5, 6]. Accordingly, there is currently no general consensus regarding how to treat or prevent acute radiation dermatitis, and practices vary widely between different hospitals and also between clinicians. In addition, some of the treatment modalities are based only on anecdotal evidence.

Adlay (*Coix lacryma-jobi* L. var.* ma-yuen* Stapf), also known as Job's tears, is an annual cereal crop which belongs to the family Gramineae. It is mainly planted in certain Asian countries including China, Japan, and India. The adlay seed is composed of four different parts from the outside to the inside, which are the hull, testa, bran, and endosperm. This cereal crop has previously been used for centuries in traditional Chinese medicine and as a food supplement. Previous studies have shown that adlay (either the whole seed or the bran part) has multiple pharmacological properties, including anti-inflammatory [7–9], antioxidant [10–12], and anticancer activities [13, 14]. In this prospective, randomized, double-blind controlled study, we seek to evaluate the efficacy of prophylactic oral therapy with adlay bran extract in reducing the risk of severe acute radiation dermatitis in patients with breast cancer undergoing radiotherapy.

## 2. Patients and Methods

### 2.1. Patient Recruitment

This is a prospective, randomized, double-blind controlled study performed at Kaohsiung Medical University Hospital. From December 2011 to August 2013, consecutive eligible female patients with unilateral breast cancer attending the Department of Radiation Oncology were asked to participate in this study. All patients received either breast conserving surgery or modified radical mastectomy followed by adjuvant radiation therapy. The exclusion criteria for this clinical trial were patients with recurrent breast cancer or distant metastases from breast cancer, pregnant women, patients receiving chemotherapy and radiotherapy at the same time, concurrent treatment with oral corticosteroids, prior radiation therapy to the breast or chest wall, past breast implants or reconstructions, and systemic connective tissue diseases (including scleroderma and lupus erythematosus). This clinical trial was approved by the ethics committee of our hospital, and all patients gave informed consent before participation in this study.

### 2.2. Radiotherapy

Adjuvant radiotherapy was administered following surgery for breast cancer. Planning for radiation therapy was performed using the Pinnacle three-dimensional treatment planning system. Each patient underwent computed tomography imaging from the level of the neck to the upper abdomen. The target volumes were defined according to the guidelines of the International Commission on Radiation Units and Measurements (ICRU) Reports 50 and 62 [15, 16]. The ipsilateral breast or chest wall (and regional lymph nodes) represented the clinical target volumes (CTV). To delineate the planning target volumes (PTV), a 5–10 mm margin was added around the CTV, to account for differences in radiotherapy treatment setup and movement during breathing.

Patients who underwent breast conserving surgery were given external beam irradiation with photons to the entire breast. Patients who underwent modified radical mastectomy received irradiation with photons to the chest wall and regional lymph nodes. The total dose of radiotherapy delivered was 50.0–50.4 Gy, administered in 1.8–2.0 Gy fractions, five days a week. The radiation dose was prescribed to a point in the midplane of the breast or chest wall (defined as the ICRU reference point). Radiotherapy was administered using two opposed tangential 6 MV photon beams, with wedges and/or up to four small subbeams to obtain a homogeneous dose distribution. The radiation dose distribution of the PTV was designed to meet the ICRU dose uniformity guidelines. Patients who underwent breast conserving surgery received an additional 10–14 Gy boost dose to the tumor bed with three-dimensional photon beam technique. The total duration of radiotherapy was 5 to 6 weeks.

### 2.3. Preparation of Adlay Bran Extract

Adlay seed samples were obtained from farmers (in Taichung, Taiwan) who planted Taichung Shuenyu number 4 (TCS4) of* Coix lacryma-jobi* L. var.* ma-yuen* Stapf. The bran part of adlay was separated from the other parts of adlay seed, protected from light, and extracted with ethanol (1 : 6; weight/volume) at room temperature for 24 hours. The plant residues were removed by centrifugation, and a rotary vacuum evaporator was employed to concentrate the ethanolic extract under reduced pressure. Around 10 grams of adlay bran was required to yield one gram of adlay bran extract. The adlay bran extract was subsequently manufactured into capsules (500 mg weight) for clinical use.

### 2.4. Patient Randomization and Treatment Schedule

Patients were randomly allocated to one of two groups (adlay bran extract or olive oil) in a double-blind fashion in a 2 : 1 ratio using a computer-generated randomization list. We had elected to use unequal randomization ratios in this study in order to increase patient acceptability of the trial and therefore improve recruitment rates and because increased number of patients being allocated to the adlay group will allow us to better monitor any possible side effects which may arise from this new treatment [17]. The adlay bran extract and olive oil oral capsules were identical in appearance with similar consistency and taste and were placed in identical-appearing boxes labeled with a code for each patient. The allocation code for each patient was kept secret in a computer file until the end of the study. The drug contents were not revealed to the clinicians and investigators until completion of the study.

Patients were instructed to take the assigned drug, four capsules a day in two divided doses (each capsule 500 mg), from the first day of radiotherapy, and to continue till the last day of radiotherapy. The total treatment period was 5-6 weeks. Patients were asked not to take oral anti-inflammatory medications or apply topical skin agents during the study period. Compliance to study medications was monitored at every follow-up visit. Patients who failed to take the assigned drug for ≥5 days were removed from the study for noncompliance.

### 2.5. Clinical Scoring Criteria for the Assessment of Acute Radiation Dermatitis

Evaluation of radiation-induced skin injury with clinical scoring criteria was performed before the start of radiotherapy and at the end of radiotherapy (on the last day of radiotherapy, i.e., 5-6 weeks after the first day of radiation therapy). Acute radiation dermatitis was graded using the Radiation Therapy Oncology Group (RTOG) scoring criteria. The acute radiation skin reactions were classified as mild (grade 1 and below) or severe (grade 2 and above). All patients were assessed jointly by an experienced radiation oncologist and a dermatologist who were blinded to the intervention received by the patients, and a consensus score for the skin reaction was determined.

### 2.6. Statistical Analysis

The data were presented as mean ± standard deviation (SD). The two-sample *t*-test was used to compare continuous variables. The Chi-square test or Fisher's exact test was employed to analyze categorical variables. Multivariate analysis to evaluate potential prognostic factors (age, body mass index, diabetes mellitus, hypertension, hemoglobin level, creatinine level, type of surgery, chemotherapy before radiotherapy, and hormone therapy) for the development of severe acute radiation dermatitis was performed using logistic regression. Statistical analysis was performed using SPSS version 19.0 (SPSS Inc., Chicago, IL, USA). Results were considered to be statistically significant if the *P* value was <0.05.

## 3. Results

### 3.1. Clinical Features of Patients

**Figure 1:**
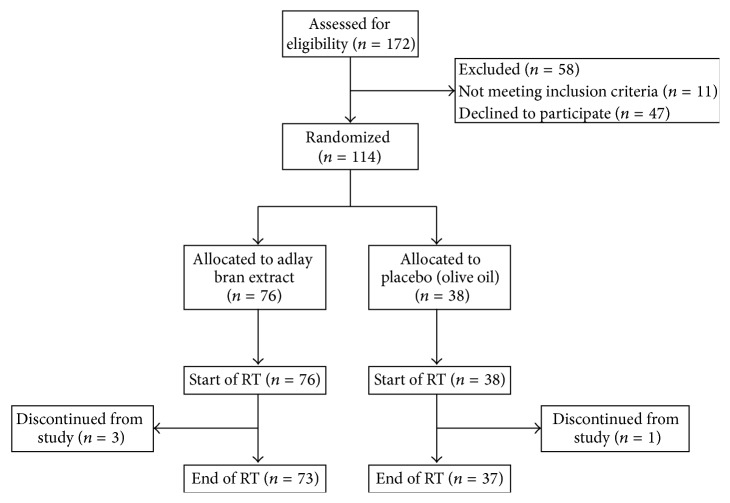
Flowchart of the participants' progress through the randomized, double-blind, clinical trial. RT: radiotherapy.

During the patient recruitment process, 172 patients were assessed for eligibility, and 58 patients were excluded (11 patients not meeting inclusion criteria and 47 patients declined to participate). The remaining 114 patients were randomly allocated in a 2 : 1 ratio to treatment with adlay bran extract or olive oil (placebo) (Figure 1). Three patients from the adlay bran extract group and one patient from the olive oil group subsequently discontinued from the study because of inadequate adherence to treatment. Consequently, 73 patients from the adlay bran extract group and 37 patients from the olive oil group completed the study. Therefore, a total of 110 women who received radiotherapy for breast cancer were analyzed in this study.

Demographic and clinical characteristics for the intervention (adlay bran extract) and placebo (olive oil) groups including age, body mass index (BMI), education, marital status, annual income, breast cancer stage, the frequency of various comorbidities (diabetes mellitus and hypertension), pretreatment blood tests (hemoglobin, creatinine, cholesterol, and triglyceride), and different treatments for breast cancer (type of surgery, chemotherapy before radiotherapy, and hormone therapy) are shown in Table 1. The adlay and placebo groups were well balanced, with no statistically significant differences with respect to patient- and treatment-related factors.

### 3.2. Comparison of Acute Radiation Dermatitis Severity between the Intervention and Placebo Groups Using RTOG Criteria

The RTOG criteria were used to grade the severity of acute radiation dermatitis in patients with breast cancer. Before the start of radiation treatment, no patients exhibited clinically obvious erythema on the irradiation field, and all patients were scored as 0 on the RTOG scale. The severity of acute radiation dermatitis (assessed using the RTOG criteria) following radiotherapy is shown in Table 2. For patients in the intervention group (treated with adlay bran extract), the proportion of patients who developed various grades of acute radiation dermatitis was as follows: 5.5% (grade 0), 49.3% (grade 1), 34.2% (grade 2), and 11.0% (grade 3). For patients treated with placebo (olive oil), the proportion of patients who developed various grades of acute radiation dermatitis was as follows: 0.0% (grade 0), 24.3% (grade 1), 67.6% (grade 2), and 8.1% (grade 3). No patients in either the intervention or placebo groups developed grade 4 acute radiation dermatitis. There was a statistically significant inverse association between adlay bran extract treatment and acute radiation dermatitis severity (*P* = 0.006, Fisher's exact test).

In addition, when patients were grouped into those who developed mild radiation dermatitis (grade 1 and below) and severe radiation dermatitis (grade 2 and above), we found that the occurrence of severe radiation dermatitis (RTOG grade ≥ 2) was significantly lower in patients treated with oral adlay bran extract compared to placebo (45.2% versus 75.7%, *P* = 0.002, Chi-square test).

### 3.3. Adverse Effects

The great majority of patients did not report any adverse effects from oral ingestion of adlay bran extract throughout the treatment period. However, one patient reported abdominal bloating, and one patient reported mild watery stools following intake of adlay bran extract. These effects were mild and did not stop the patients from continuing to take the medications. No serious adverse reactions were noted.

### 3.4. Patient- and Treatment-Related Factors for the Development of Severe Acute Radiation Dermatitis

Potential patient- and treatment-related factors which may be associated with the development of severe acute radiation dermatitis (RTOG grade ≥ 2) were analyzed, including age, body mass index, diabetes, hypertension, hemoglobin level, creatinine level, type of surgery, chemotherapy before radiotherapy, and hormone therapy. In the univariate analysis, body mass index was found to be significantly associated with the development of severe acute radiation dermatitis (Table 3). Multivariate analysis using logistic regression showed that the risk of developing severe acute radiation dermatitis (grade 2 or higher) was increased for patients with higher body mass index (adjusted odds ratio 1.31, *P* = 0.002) and decreased for patients treated with adlay bran extract compared to placebo (adjusted odds ratio 0.24, *P* = 0.004) (Table 4). Patients with diabetes also appeared to have higher risk of developing severe acute radiation dermatitis (adjusted odds ratio 3.95), but the trend did not reach statistical significance (*P* = 0.172). On the other hand, the risk of severe acute radiation dermatitis was not significantly altered by age, hypertension, hemoglobin level, creatinine level, type of surgery, chemotherapy before radiotherapy, or hormone therapy.

## 4. Discussion

Acute radiation dermatitis is a common side effect in patients with breast cancer receiving radiation therapy. Although some patients develop only mild skin erythema, the skin reaction in a subset of patients may progress to moist desquamation and ulceration. Acute radiation-induced skin damage may have major adverse impacts on a patient's well-being and quality of life [18]. In severe cases, radiotherapy may have to be interrupted or terminated, which may have negative consequences for cancer control and treatment [19].

Currently, the mechanism of skin injury due to ionizing radiation is only partially understood. Acute radiation exposure causes single- and double-stranded DNA breaks and induces direct injury to cells of the epidermis and hair follicle (particularly stem cells), dermal fibroblasts, and endothelial cells [2, 20]. Furthermore, it leads to the generation of reactive oxygen species, which may cause damage to cellular DNA, proteins, and lipids. In addition, radiation-associated skin injury leads to the recruitment of inflammatory cells and production of cytokines, resulting in skin inflammation [1, 21].

Various clinical scoring systems have been developed to grade the severity of acute radiation dermatitis, the most common of which are the Radiation Therapy Oncology Group (RTOG) and the Common Terminology Criteria for Adverse Events (CTCAE) criteria [22–24]. The RTOG criteria are widely applied in clinical and research settings and have been demonstrated to have good intraobserver and interobserver agreement [25] and are therefore used in this study.

Although acute radiation dermatitis is a frequent adverse effect of radiotherapy, there is currently no standard management for this condition [26]. There have been only a small number of studies providing evidence-based interventions for radiation-induced skin reactions, and some of the reports are conflicting. Topical corticosteroids were found by some studies to be beneficial in preventing or treating acute radiation dermatitis [27–29], but other studies showed no positive effect [30]. Moreover, the application of topical steroids may lead to thinning of skin and increase the risk of bacterial infections. Topical aloe vera was frequently used for radiation-induced skin reactions but was shown not to be effective in a number of studies [31]. Calendula cream was demonstrated by one study to decrease the risk of acute radiation dermatitis of grade 2 or higher [32] but was found to have no clear benefit in another study [33]. The evidence for topical hyaluronic acid was also conflicting, with some studies showing it to be effective [34, 35], while others demonstrated that it may actually be detrimental [36].

It is therefore clear that there is insufficient evidence in the literature to support the use of any single agent for the prevention or treatment of acute radiation dermatitis [5, 6, 37]. Limited and often conflicting evidence for the care of radiation skin reactions is associated with large variations in the clinical management of this condition between different institutions and also between individual clinicians [38, 39]. In addition, a substantial number of recommended interventions are based only on anecdotal evidence. Therefore, there is a clear need for further studies to identify new agents for the prevention or treatment of radiation dermatitis.

Adlay seed had been previously shown to have anti-inflammatory and antioxidant properties. In particular, the bran part of adlay had been demonstrated to have greater anti-inflammatory and antioxidant activities compared to other parts of adlay seed [12]. Since ionizing radiation induces an acute inflammatory response in the skin with production of reactive oxygen species, we hypothesize that oral prophylactic treatment with adlay bran extract may ameliorate the severity of acute radiation dermatitis in patients with breast cancer undergoing radiotherapy. The findings of this study showed that the occurrence of severe radiation dermatitis (RTOG grade ≥ 2) was significantly lower in patients who received adlay bran extract treatment compared to patients who received placebo (45.2% versus 75.7%, adjusted odds ratio 0.24).

In this study, we had elected to use unequal randomization ratios (2 : 1 in favor of the experimental group) to increase patient acceptability of the trial and therefore improve recruitment rates and because increased number of patients being allocated to the adlay group will allow us to better monitor any possible side effects which may arise from this new treatment. Since the treatment allocation process is entirely random, the use of unequal group sizes in this clinical trial is not expected to affect the validity of our data. In fact, some investigators have argued that unequal randomization has been underutilized in the design of clinical trials and recommended that it should be used more often in appropriate situations [17]. In this study, we have chosen to use olive oil as the placebo because it has identical appearance as the adlay bran extract with similar consistency and taste. This enabled double-blinding to be achieved in this clinical trial.

In the present study, the occurrence rate of severe acute radiation dermatitis (RTOG grade ≥ 2) was higher compared to previous reports [25, 32, 33]. This may possibly be due to ethnic differences in radiation-induced skin reactions, variations in radiotherapy technique between different institutions, differences in clinical management of radiation dermatitis (patients in this study were asked not to take oral anti-inflammatory medications or apply topical skin agents), and the subjectivity of the RTOG clinical scoring criteria (radiation-induced skin reaction in a particular patient may be graded as either “faint” or “bright” erythema by different investigators). Since all patients in our study were assessed jointly by the same radiation oncologist and dermatologist, the RTOG grades of patients can be compared within this study but not with other studies.

Adlay bran contains a substantial amount of neutral oil (around 25% of the dry weight) [40]. Fatty acids that are present in greatest amounts in adlay bran are oleic acid, linoleic acid, palmitic acid, and stearic acid. In addition, significant amounts of phytosterols, phenolic compounds, and flavonoids are present in the adlay bran [11]. Although the exact active components in adlay bran are currently unknown, phenolic compounds and flavonoids had been shown to contribute to the antioxidant and anti-inflammatory actions of adlay bran [8, 9, 12]. In terms of molecular mechanisms of action, adlay bran had been demonstrated to exert anti-inflammatory effects through suppression of COX-2 expression [7] and inhibition of nitric oxide production [8]. In addition, adlay bran had been shown to mediate antioxidant activity through scavenging of superoxide anion radicals [12]. Further investigations are required to identify the biologically active constituents of adlay bran and elucidate the molecular mechanisms for their possible anti-inflammatory and antioxidant actions in relation to acute radiation dermatitis.

Previously, adlay seed has been used for centuries in certain Asian countries as a food supplement without obvious adverse effects. In our study, the great majority of patients also did not report any side effects from oral ingestion of adlay bran extract throughout the treatment period. This indicates that adlay bran extract may be a safe form of treatment for acute radiation dermatitis.

In terms of patient- and treatment-related factors, multivariate analysis in our study showed body mass index to be a significant prognostic factor for the development of severe acute radiation dermatitis (RTOG grade ≥ 2). This is in agreement with a previous study demonstrating an association between higher body mass index and acute radiation dermatitis of grade 2 or higher [32]. On the other hand, the risk of severe acute radiation dermatitis was not significantly altered by age, hypertension, hemoglobin level, creatinine level, type of surgery, chemotherapy before radiotherapy, or hormone therapy in our study. Previous studies in patients with breast cancer receiving radiotherapy had also demonstrated no significant associations between the severity of acute radiation dermatitis and patient characteristics including diabetes, hypertension, previous chemotherapy, and hormone therapy [36, 41, 42].

In summary, the results of this prospective, randomized, double-blind study indicate that oral prophylactic therapy with adlay bran extract may reduce the risk of acute radiation dermatitis of grade 2 or higher in patients with breast cancer undergoing radiotherapy. No serious adverse effects due to adlay bran treatment were noted. Therefore, adlay bran extract may have potential use in the future for the prevention of severe acute radiation skin reactions. Further clinical studies with larger numbers of patients will be required to determine the optimal dosage and duration of administration for patients receiving radiotherapy.

## Figures and Tables

**Table 1:** Demographic and clinical characteristics of patients in the adlay bran extract and placebo groups.

	Adlay bran group (*n* = 73)	Placebo group (*n* = 37)	*P* value
Age (mean ± SD)	51.2 ± 10.5	52.1 ± 9.2	0.672
Body mass index (mean ± SD)	22.4 ± 3.2	23.2 ± 2.8	0.247
Education			0.933
Junior high school or lower	19 (26.0%)	9 (24.3%)	
Senior high school	25 (34.2%)	14 (37.8%)	
College or above	29 (39.7%)	14 (37.8%)	
Marital status			0.822
Single	9 (12.3%)	4 (10.8%)	
Married	50 (68.5%)	24 (64.9%)	
Widowed or divorced	14 (19.2%)	9 (24.3%)	
Annual income (TWD)			0.329
<400,000	30 (41.1%)	13 (35.1%)	
400,000–800,000	16 (21.9%)	13 (35.1%)	
>800,000	27 (37.0%)	11 (29.7%)	
Breast cancer stage			0.326
0	11 (15.1%)	3 (8.1%)	
I	30 (41.1%)	17 (45.9%)	
II	15 (20.5%)	12 (32.4%)	
III	17 (23.3%)	5 (13.5%)	
Diabetes mellitus			0.730
Yes	6 (8.2%)	4 (10.8%)	
No	67 (91.8%)	33 (89.2%)	
Hypertension			1.000
Yes	9 (12.3%)	5 (13.5%)	
No	64 (87.7%)	32 (86.5%)	
Pretreatment hemoglobin level	11.99 ± 1.26	11.91 ± 1.29	0.748
Pretreatment creatinine level	0.734 ± 0.908	0.628 ± 0.114	0.482
Pretreatment fasting cholesterol level	193.1 ± 37.0	195.7 ± 45.0	0.750
Pretreatment fasting triglyceride level	113.3 ± 78.9	136.9 ± 104.1	0.188
Surgery			0.190
Breast conserving surgery	61 (83.6%)	27 (73.0%)	
Modified radical mastectomy	12 (16.4%)	10 (27.0%)	
Chemotherapy before radiotherapy			0.458
Yes	42 (57.5%)	24 (64.9%)	
No	31 (42.5%)	13 (35.1%)	
Hormone therapy			0.522
Yes	51 (69.9%)	28 (75.7%)	
No	22 (30.1%)	9 (24.3%)	

*P* values were determined using the two-sample *t*-test for continuous variables and the Chi-square or Fisher's exact test for categorical variables.

TWD: Taiwan Dollar (1 Taiwan Dollar = 0.0325 United States Dollar).

**Table 2:** Comparison of acute radiation dermatitis severity in the intervention (adlay bran extract) and placebo (olive oil) groups assessed using the RTOG criteria.

RTOG grade	Adlay bran extract (*n* = 73)	Placebo (*n* = 37)	*P* value
Skin reaction			0.006
Grade 0	4 (5.5%)	0 (0.0%)	
Grade 1	36 (49.3%)	9 (24.3%)	
Grade 2	25 (34.2%)	25 (67.6%)	
Grade 3	8 (11.0%)	3 (8.1%)	
Grade 4	0 (0.0%)	0 (0.0%)	
Skin reaction			0.002
Grade ≤ 1	40 (54.8%)	9 (24.3%)	
Grade ≥ 2	33 (45.2%)	28 (75.7%)	

*P* values were determined by the Chi-square or Fisher's exact test.

**Table 3:** Univariate analysis to determine prognostic factors for the development of severe acute radiation dermatitis.

Factor	Skin reaction (RTOG)		*P* value
	Grade ≤ 1 (*n* = 49)	Grade ≥ 2 (*n* = 61)	
Age	50.3 ± 9.5	52.5 ± 10.4	0.246
Body mass index	21.6 ± 2.3	23.5 ± 3.3	<0.001
Diabetes mellitus			0.180
Yes	2 (4.1%)	8 (13.1%)	
No	47 (95.9%)	53 (86.9%)	
Hypertension			0.477
Yes	5 (10.2%)	9 (14.8%)	
No	44 (89.8%)	52 (85.2%)	
Pretreatment hemoglobin level	11.90 ± 1.24	12.01 ± 1.29	0.654
Pretreatment creatinine level	0.618 ± 0.178	0.764 ± 0.983	0.307
Surgery			0.565
Breast conserving surgery	38 (77.6%)	50 (82.0%)	
Modified radical mastectomy	11 (22.4%)	11 (18.0%)	
Chemotherapy			0.531
Yes	31 (63.3%)	35 (57.4%)	
No	18 (36.7%)	26 (42.6%)	
Hormone therapy			0.612
Yes	34 (69.4%)	45 (73.8%)	
No	15 (30.6%)	16 (26.2%)	

*P* values were determined using the two-sample *t*-test for continuous variables and the Chi-square or Fisher's exact test for categorical variables.

**Table 4:** Multivariate analysis to determine prognostic factors for the development of severe acute radiation dermatitis (RTOG grade ≥ 2) (*n* = 110).

Factor	Adjusted odds ratio	95% CI	*P* value
Age	1.00	0.95–1.05	0.964
Body mass index	1.31	1.11–1.56	0.002
Diabetes mellitus	3.95	0.55–28.27	0.172
Hypertension	0.96	0.20–4.62	0.955
Pretreatment hemoglobin level	0.91	0.59–1.38	0.645
Pretreatment creatinine level	1.77	0.51–6.12	0.368
Surgery (MRM versus BCS)	0.82	0.26–2.60	0.736
Chemotherapy before radiotherapy	0.44	0.13–1.49	0.189
Hormone therapy	1.02	0.39–2.69	0.963
Intervention (adlay bran versus placebo)	0.24	0.09–0.63	0.004

Multivariate analysis was performed using logistic regression.

MRM: modified radical mastectomy, BCS: breast conserving surgery, and CI: confidence interval.

## References

[1] Ryan J. L. (2012). Ionizing radiation: the good, the bad, and the ugly. *The Journal of Investigative Dermatology*.

[2] Hymes S. R., Strom E. A., Fife C. (2006). Radiation dermatitis: clinical presentation, pathophysiology, and treatment 2006. *Journal of the American Academy of Dermatology*.

[3] Harper J. L., Franklin L. E., Jenrette J. M., Aguero E. G. (2004). Skin toxicity during breast irradiation: pathophysiology and management. *Southern Medical Journal*.

[4] Brown K. R., Rzucidlo E. (2011). Acute and chronic radiation injury. *Journal of Vascular Surgery*.

[5] Salvo N., Barnes E., van Draanen J. (2010). Prophylaxis and management of acute radiation-induced skin reactions: a systematic review of the literature. *Current Oncology*.

[6] Bolderston A., Lloyd N. S., Wong R. K., Holden L., Robb-Blenderman L. (2006). The prevention and management of acute skin reactions related to radiation therapy: a systematic review and practice guideline. *Supportive Care in Cancer*.

[7] Chung C.-P., Hsu H.-Y., Huang D.-W. (2010). Ethyl acetate fraction of adlay bran ethanolic extract inhibits oncogene expression and suppresses DMH-induced preneoplastic lesions of the colon in F344 rats through an anti-inflammatory pathway. *Journal of Agricultural and Food Chemistry*.

[8] Huang D.-W., Wu C.-H., Shih C.-K. (2014). Application of the solvent extraction technique to investigation of the anti-inflammatory activity of adlay bran. *Food Chemistry*.

[9] Chen H.-J., Chung C.-P., Chiang W., Lin Y.-L. (2011). Anti-inflammatory effects and chemical study of a flavonoid-enriched fraction from adlay bran. *Food Chemistry*.

[10] Chung C.-P., Hsia S.-M., Lee M.-Y. (2011). Gastroprotective activities of adlay (*Coix lachryma-jobi L. var. ma-yuen* Stapf) on the growth of the stomach cancer AGS cell line and indomethacin-induced gastric ulcers. *Journal of Agricultural and Food Chemistry*.

[11] Yao H.-T., Lin J.-H., Chiang M.-T., Chiang W., Luo M.-N., Lii C.-K. (2011). Suppressive effect of the ethanolic extract of adlay bran on cytochrome P-450 enzymes in rat liver and lungs. *Journal of Agricultural and Food Chemistry*.

[12] Zhao M., Zhu D., Sun-Waterhouse D. (2014). In vitro and in vivo studies on adlay-derived seed extracts: phenolic profiles, antioxidant activities, serum uric acid suppression, and xanthine oxidase inhibitory effects. *Journal of Agricultural and Food Chemistry*.

[13] Chung C.-P., Hsu C.-Y., Lin J.-H., Kuo Y.-H., Chiang W., Lin Y.-L. (2011). Antiproliferative lactams and spiroenone from adlay bran in human breast cancer cell lines. *Journal of Agricultural and Food Chemistry*.

[14] Lee M.-Y., Lin H.-Y., Cheng F., Chiang W., Kuo Y.-H. (2008). Isolation and characterization of new lactam compounds that inhibit lung and colon cancer cells from adlay (*Coix lachryma-jobi L. var. ma-yuen* Stapf) bran. *Food and Chemical Toxicology*.

[15] International Commission on Radiation Units and Measurements (ICRU) (1993). Prescribing, recording, and reporting photon beam therapy. *ICRU Report*.

[16] International Commission on Radiation Units and Measurements (ICRU) (1999). *ICRU Report 62: Prescribing, Recording and Reporting Photon Beam Therapy (Supplement to ICRU Report 50)*.

[17] Dumville J. C., Hahn S., Miles J. N. V., Torgerson D. J. (2006). The use of unequal randomisation ratios in clinical trials: a review. *Contemporary Clinical Trials*.

[18] Schnur J. B., Ouellette S. C., Dilorenzo T. A., Green S., Montgomery G. H. (2011). A qualitative analysis of acute skin toxicity among breast cancer radiotherapy patients. *Psycho-Oncology*.

[19] Bese N. S., Hendry J., Jeremic B. (2007). Effects of prolongation of overall treatment time due to unplanned interruptions during radiotherapy of different tumor sites and practical methods for compensation. *International Journal of Radiation Oncology, Biology, Physics*.

[20] Denham J. W., Hauer-Jensen M. (2002). The radiotherapeutic injury—a complex ‘wound’. *Radiotherapy and Oncology*.

[21] Müller K., Meineke V. (2007). Radiation-induced alterations in cytokine production by skin cells. *Experimental Hematology*.

[22] Cox J. D., Stetz J., Pajak T. F. (1995). Toxicity criteria of the Radiation Therapy Oncology Group (RTOG) and the European Organization for Research and Treatment of Cancer (EORTC). *International Journal of Radiation Oncology, Biology, Physics*.

[23] Dische S. (1994). The uniform reporting of treatment-related morbidity. *Seminars in Radiation Oncology*.

[24] Trotti A., Colevas A. D., Setser A. (2003). CTCAE v3.0: development of a comprehensive grading system for the adverse effects of cancer treatment. *Seminars in Radiation Oncology*.

[25] López E., Núñez M. I., Guerrero M. R. (2002). Breast cancer acute radiotherapy morbidity evaluated by different scoring systems. *Breast Cancer Research and Treatment*.

[26] Feight D., Baney T., Bruce S., McQuestion M. (2011). Putting evidence into practice: evidence-based interventions for radiation dermatitis. *Clinical Journal of Oncology Nursing*.

[27] Boström Å., Lindman H., Swartling C., Berne B., Bergh J. (2001). Potent corticosteroid cream (mometasone furoate) significantly reduces acute radiation dermatitis: results from a double-blind, randomized study. *Radiotherapy and Oncology*.

[28] Schmuth M., Wimmer M. A., Hofer S. (2002). Topical corticosteroid therapy for acute radiation dermatitis: a prospective, randomized, double-blind study. *British Journal of Dermatology*.

[29] Miller R. C., Schwartz D. J., Sloan J. A. (2011). Mometasone furoate effect on acute skin toxicity in breast cancer patients receiving radiotherapy: a phase III double-blind, randomized trial from the North Central Cancer Treatment Group N06C4. *International Journal of Radiation Oncology Biology Physics*.

[30] Potera M. E., Lookingbill D. P., Stryker J. A. (1982). Prophylaxis of radiation dermatitis with a topical cortisone cream. *Radiology*.

[31] Richardson J., Smith J. E., McIntyre M., Thomas R., Pikington K. (2005). Aloe vera for preventing radiation-induced skin reactions: a systematic literature review. *Clinical Oncology*.

[32] Pommier P., Gomez F., Sunyach M. P., D'Hombres A., Carrie C., Montbarbon X. (2004). Phase III randomized trial of Calendula Officinalis compared with trolamine for the prevention of acute dermatitis during irradiation for breast cancer. *Journal of Clinical Oncology*.

[33] Sharp L., Finnilä K., Johansson H., Abrahamsson M., Hatschek T., Bergenmar M. (2013). No differences between Calendula cream and aqueous cream in the prevention of acute radiation skin reactions—results from a randomised blinded trial. *European Journal of Oncology Nursing*.

[34] Liguori V., Guillemin C., Pesce G. F., Mirimanoff R. O., Bernier J. (1997). Double-blind, randomized clinical study comparing hyaluronic acid cream to placebo in patients treated with radiotherapy. *Radiotherapy & Oncology*.

[35] Leonardi M. C., Gariboldi S., Ivaldi G. B. (2008). A double-blind, randomised, vehicle-controlled clinical study to evaluate the efficacy of MAS065D in limiting the effects of radiation on the skin: interim analysis. *European Journal of Dermatology*.

[36] Pinnix C., Perkins G. H., Strom E. A. (2012). Topical hyaluronic acid vs. standard of care for the prevention of radiation dermatitis after adjuvant radiotherapy for breast cancer: single-blind randomized phase III clinical trial. *International Journal of Radiation Oncology Biology, Physics*.

[37] Chan R. J., Larsen E., Chan P. (2012). Re-examining the evidence in radiation dermatitis management literature: an overview and a critical appraisal of systematic reviews. *International Journal of Radiation Oncology Biology Physics*.

[38] Kumar S., Juresic E., Barton M., Shafiq J. (2010). Management of skin toxicity during radiation therapy: a review of the evidence. *Journal of Medical Imaging and Radiation Oncology*.

[39] D'Haese S., Bate T., Claes S., Boone A., VanVoorden V., Efficace F. (2005). Management of skin reactions during radiotherapy: a study of nursing practice. *European Journal of Cancer Care*.

[40] Huang B.-W., Chiang M.-T., Yao H.-T., Chiang W. (2005). The effect of adlay oil on plasma lipids, insulin and leptin in rat. *Phytomedicine*.

[41] Morganti A. G., Digesù C., Panunzi S. (2009). Radioprotective effect of moderate wine consumption in patients with breast carcinoma. *International Journal of Radiation Oncology, Biology, Physics*.

[42] Hijal T., Al Hamad A. A., Niazi T. (2010). Hypofractionated radiotherapy and adjuvant chemotherapy do not increase radiation-induced dermatitis in breast cancer patients. *Current Oncology*.

